# The Interactions of the Complement System with Human Cytomegalovirus

**DOI:** 10.3390/v16071171

**Published:** 2024-07-20

**Authors:** Eduardo Lujan, Isadora Zhang, Andrea Canto Garon, Fenyong Liu

**Affiliations:** 1 Program in Comparative Biochemistry, University of California, Berkeley, CA 94720, USA; 2 School of Public Health, University of California, Berkeley, CA 94720, USA

**Keywords:** herpesvirus, cytomegalovirus, complement, innate immunity

## Abstract

The complement system is an evolutionarily ancient component of innate immunity that serves as an important first line of defense against pathogens, including viruses. In response to infection, the complement system can be activated by three distinct yet converging pathways (classical, lectin, and alternative) capable of engaging multiple antiviral host responses to confront acute, chronic, and recurrent viral infections. Complement can exert profound antiviral effects via multiple mechanisms including the induction of inflammation and chemotaxis to sites of infection, neutralization/opsonization of viruses and virally infected cells, and it can even shape adaptive immune responses. With millions of years of co-evolution and the ability to establish life-long infections, herpesviruses have evolved unique mechanisms to counter complement-mediated antiviral defenses, thus enabling their survival and replication within humans. This review aims to comprehensively summarize how human herpesviruses engage with the complement system and highlight our understanding of the role of complement in human cytomegalovirus (HCMV) infection, immunity, and viral replication. Herein we describe the novel and unorthodox roles of complement proteins beyond their roles in innate immunity and discuss gaps in knowledge and future directions of complement and HCMV research.

## 1. The Complement System

The human complement system is a crucial sentinel in the battle against infection, representing an ancient evolutionary branch of innate immunity with homologs identified in diverse life forms including invertebrates [1], insects [2], and even organisms of lower complexity [3]. In humans, the complement system comprises approximately 30 proteins predominantly synthesized by the liver and found in high concentrations in serum, or expressed on cell surfaces by diverse cell types [4]. While typically inactive, complement proteins are rapidly activated upon infection or injury in a meticulously coordinated sequence of proteolytic and enzymatic cascading reactions. The multiple outcomes of this highly regulated and orchestrated response include the following: (i) inflammation, (ii) pathogen membrane lysis, (iii) opsonization, and (iv) the recruitment of immune cells to sites of infection. The complement system is composed of three distinct pathways: the classical pathway, the lectin pathway, and the alternative pathway. The activation of each complement pathway can occur independently or concomitantly, depending on distinct molecular cues. Irrespective of which complement pathway is activated, all three pathways converge at the central complement component 3 (C3) [5].

### 1.1. The Classical Pathway 

**Figure 1:**
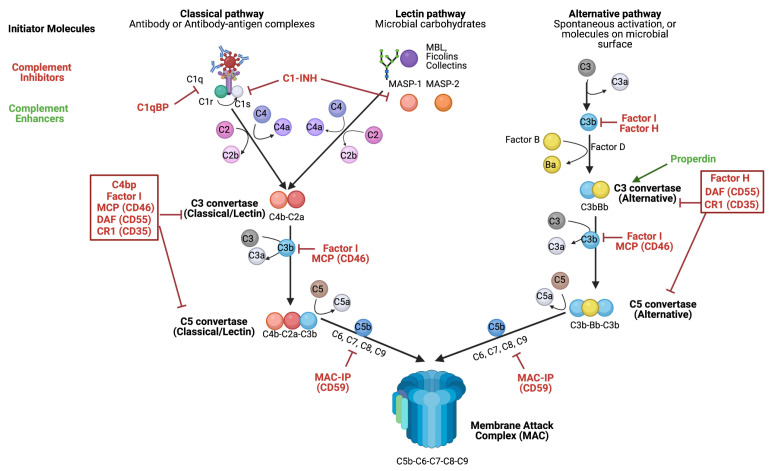
Overview of the human complement system. The complement system comprises three distinct, yet converging pathways: the classical, the lectin, and the alternative. The activation of each complement pathway generally requires unique molecular cues (initiator molecules) for initiation and depends on several host proteases for continuation to subsequent steps in the complement cascade. The proteolytic cleavage of complement proteins yields biproducts that assemble into multimolecular complexes with enzymatic activity known as C3 and C5 convertases, both of which contribute to amplification and the terminal stage of the complement cascade referred to as the membrane attack complex (MAC). The MAC induces a membrane pore with potent lytic activity capable of neutralizing diverse microorganisms. To prevent self-damage, the complement system is tightly regulated in all three pathways at multiple levels by soluble and membrane-bound host complement inhibitors (red), and to a lesser degree, host complement enhancers (green). Original figure designed with the assistance of Biorender software (www.biorender.com). See references [5,6,7].

As illustrated in Figure 1, the classical pathway of complement activation commences with the recognition of antibody or antibody–antigen complexes by the C1 complex. The C1 complex adopts a distinctive structure of C1q-(C1r^2^-C1s^2^), comprised of a single molecule of C1q and a heterotetrameric protease complex consisting of two molecules each of C1r and C1s [6]. Within this complex, C1q mediates pattern recognition by binding to the Fc domain of IgM and IgG antibody [6], while the C1r and C1s subunits function as serine proteases with narrow specificity and minimal enzymatic activity [6]. Upon C1q-antibody binding, the C1 complex is activated, resulting in the autoactivation of C1r, which in turn cleaves and activates C1s. Activated C1s subsequently cleaves and activates complement component 4 (C4) and complement component 2 (C2). C1s cleaves C4 into C4a and C4b, whereby C4b serves as an opsonin by binding directly to complement activating surfaces [5]. Additionally, C1s cleaves C2 into C2a and C2b. C2a, along with C4b, contributes to the formation of the C3 convertase (C4bC2a) that is shared by the classical and lectin pathways [5]. This C3 convertase, as its name suggests, cleaves complement component 3 (C3) into C3a and C3b (see Figure 1). Following the cleavage of C3, C3a serves as an anaphylatoxin, inducing inflammation, chemotaxis, and extravasation of cells to sites of infection, while C3b binds to C4bC2a to generate the C5 convertase (C4bC2aC3b) shared by the classical and lectin pathways [7].

### 1.2. The Lectin Pathway 

As depicted in Figure 1, the lectin pathway of complement activation begins with the recognition of foreign carbohydrates on the surface of microorganisms by pattern recognition molecules such as the following: mannose-binding lectin (MBL), Collectins, and Ficolins. A distinguishing trait of these pattern recognition molecules within the lectin pathway is their capacity to engage with a group of serine proteases known as mannose-binding lectin (MBL)-associated serine proteases (MASPs), including MASP-1 and MASP-2. Both MASP-1 and MASP-2 mediate the activation of the lectin pathway, and like the classical pathway, results in the cleavage of C4 and C2 by MASP-1 and MASP-2 [5], with MASP-2 presumed to be the principal serine protease of the lectin pathway [8]. As in the classical pathway, C4 and C2 are cleaved to produce C4a, C4b, C2a, and C2b, resulting in the subsequent assembly of the C3 convertase (C4bC2a) and C5 convertase (C4bC2aC3b), both of which are identical in the classical and lectin pathways [5,7].

### 1.3. The Alternative Pathway

Unlike the classical and lectin pathways of complement activation, the alternative pathway can be activated by the spontaneous hydrolysis of C3 or by various proteins, lipids, and carbohydrates on microbial surfaces, as illustrated in Figure 1 [5]. Upon activation of the alternative pathway, Factor B (FB) associates with C3b and is cleaved into Bb and Ba by the serine protease Factor D (FD), generating the alternative pathway C3 convertase (C3bBb). After formation of the alternative pathway C3 convertase, Properdin/Factor P (FP) is recruited to stabilize the enzymatic structure of the C3 convertase; thus, Properdin functions as one of the few molecules that can positively regulate complement [5]. Despite its unique molecular composition, the alternative pathway C3 convertase functions akin to that of the classical and lectin pathways, cleaving C3 into C3a and C3b to generate the alternative pathway C5 convertase (C3bBbC3b) (see Figure 1). Crucially, while the alternative pathway is capable of functioning independently, it also serves as an important amplification loop for the classical and lectin pathways by synthesizing substantial amounts of C3b, a vital component for subsequent steps in the complement cascade [5].

### 1.4. The Membrane Attack Complex (MAC)

Activation of the classical, lectin, or alternative pathways of complement ultimately results in the formation of a multimolecular complex known as the C5 convertase, which precedes the terminal stage of the complement cascade. As its name implies, the C5 convertase catalyzes the cleavage of complement component 5 (C5) into the anaphylatoxin C5a, and a component of the terminal complement cascade known as C5b. While the classical and lectin pathways share a C5 convertase comprised of C4bC2aC3b, the alternative pathway has a distinct C5 convertase composed of C3bBbC3b [5]. The terminal stage of the complement cascade involves the formation of a multimolecular protein complex known as the membrane attack complex (MAC), which forms on complement-activating surfaces such as the surface of microorganisms. The MAC consists of one molecule each of the complement components C5b, C6, C7, C8, and an estimated 12–18 molecules of complement component 9 (C9), yielding an overall pore structure with a molecular architecture of C5b-C6-C7-C8-C9^12-18^ [9]. Consequently, the formation of the MAC on the membrane of microorganisms represents a potent and effective means of neutralizing pathogens by inducing microbial lysis [10]. For additional information, refer to Table 1, which lists a comprehensive inventory of major complement proteins, their primary functions, and associated pathways.

## 2. Complement in Antiviral Immunity and Mechanisms of Viral Complement Evasion

### 2.1. Antiviral Effects of Complement 

Complement can exert profound direct and indirect antiviral effects alone or in combination with other effector molecules and cells of the innate and adaptive immune system via multiple mechanisms, including the following.

#### 2.1.1. Recognition and Opsonization

Complement pattern recognition molecules recognize foreign carbohydrates (MBL, Ficolins, Collectins), antibody-coated virions (C1q), or pathogen-associated molecular patterns (PAMPs), resulting in the activation of the lectin and classical pathways that lead to the deposition of opsonins such as MBL, C3b, iC3b, and C4b on the viral envelope and surface proteins of viruses or on the membranes of virally infected cells. Opsonization in turn enhances phagocytosis by phagocytic cells, thereby facilitating the clearance of viral particles and/or infected cells [12].

#### 2.1.2. Induction of Chemotaxis to Sites of Infection and Regulating Inflammatory Cytokine Responses 

Biproducts of complement activation (C3a, C5a) act as potent anaphylatoxins capable of inducing inflammation, chemotaxis, increased vascular permeability, and the extravasation of effector immune cells (macrophages, neutrophils, etc.) to sites of viral infection [13]. Additionally, C5a (and to a lesser extent C3a) is reported to induce the expression of pro-inflammatory cytokines TNF-a and IL-1B in monocytes and macrophages, thus contributing to the regulation of local inflammatory responses [14].

#### 2.1.3. Membrane Lysis of Viruses and Virally Infected Cells 

The activation of the complement cascade generates membrane attack complexes (MACs) that assemble on the membrane surface of enveloped viruses or virally infected cells, inducing the formation of a pore that results in the loss of the infectivity of virions [5]. The lytic activity of MACs represents a potent mechanism for directly neutralizing viruses and preventing their dissemination within the host [5]. Additionally, the disruption of the viral envelope by MACs can potentially expose viral antigens, thereby promoting their recognition and clearance by the adaptive immune system [15].

#### 2.1.4. Enhancement of Antiviral Adaptive Immune Responses 

The binding of C3 and C5 cleavage products to their cognate receptors on B-cells and T-cells is reported to induce intracellular signaling pathways capable of modulating B-cell and T-cell functions. For example, the binding of C3 cleavage products (C3d, iC3b, C3dg) to complement receptor 1 (CR1/CD35) and complement receptor 2 (CR2/CD21) can enhance B-cell activation and contribute to co-stimulation [16]. Similarly, C3d-coated antigens have strong adjuvant properties and can reduce the threshold for B-cell receptor signaling [17]. As with B-cells, the complement system is also reported to contribute to the regulation of T-cell responses through membrane-bound complement regulatory proteins. For example, membrane cofactor protein (MCP), herein referred to as CD46, is expressed on T-cells, and can interact with C3b and C4b to promote T-cell activation or induce T-cell anergy, depending on the context of complement activation [18].

### 2.2. Viral Mechanisms Counteracting the Complement System 

**Figure 2:**
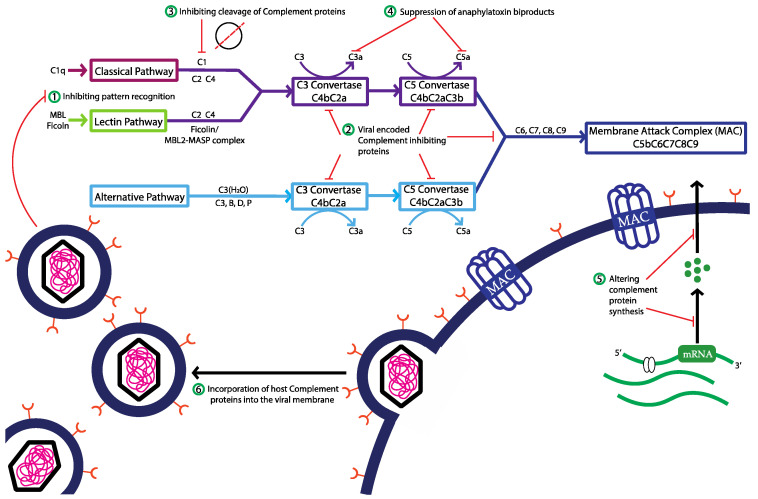
Mechanisms of complement evasion by viruses. General overview of complement evasion strategies of viruses. ① Inhibition of complement pattern recognition molecules, ② virally encoded complement inhibiting proteins, ③ inhibiting cleavage of complement proteins, ④ suppression of anaphylatoxin biproducts, ⑤ altering of complement protein synthesis during infection, and ⑥ incorporation of host complement proteins into the viral membrane. For specific examples of viruses and virally encoded proteins mediating these complement evasion strategies, see references [19,20,21,22,23,24,25,26,27,28].

A general and non-specific overview of complement evasion strategies employed by viruses to thwart the complement system is illustrated in Figure 2 (see reviews [19,20,21,22,23,24,25,26,27,28]), including the following.

#### 2.2.1. Inhibition of Complement Pattern Recognition Molecules 

Viruses can produce proteins that cleave or interfere with pattern recognition molecules (C1q, MBL) of the complement system, thus preventing the activation of the classical and lectin pathways (and to a lesser degree the alternative pathway) [24,27].

#### 2.2.2. Virally Encoded Complement Regulating Proteins 

Viruses can encode proteins that mimic host complement inhibitors (CD46, CD55, CD59), which prevent the formation or accelerate the decay of the C3 and C5 convertases in the classical, lectin, and alternative pathways of complement. Via this mechanism, viruses can globally suppress the complement cascade at the level of C3 and C5 and prevent the amplification of C3b and C5b necessary in central and terminal steps of the complement pathway, respectively [24].

#### 2.2.3. Inhibition of Cleavage of Complement Proteins 

Viruses can encode protease inhibitors that target host serine proteases that are critical in the activation and regulation of nearly all complement pathways. For example, the activation of both the classical and lectin pathways requires serine proteases, and although not directly involved in the initiation of the alternative pathway, serine proteases regulate the alternative pathway by cleaving and inactivating complement components. Thus, by inhibiting the function of host serine proteases, viruses can suppress complement activation, amplification, and terminal complement effector functions [28].

#### 2.2.4. Suppression of Anaphylatoxin Biproducts 

By inhibiting complement activation or amplification, viruses can suppress the production of complement-derived anaphylatoxins (C3a and C5a) and potentially interfere with their signaling pathways, thereby attenuating local inflammatory responses and suppressing extravasation of immune cells to sites of viral infection [29].

#### 2.2.5. Altering Complement Protein Synthesis during Infection 

Viruses are known to modulate intracellular protein synthesis and trafficking networks during infection and viral replication. By increasing or decreasing the synthesis of complement proteins during infection, viruses may suppress extracellular local complement activation and impair the neutralization and opsonization of virally infected cells [24].

#### 2.2.6. Incorporation of Host Complement Proteins into the Viral Membrane 

Viruses can resist complement-mediated lysis by incorporating host-derived complement regulatory proteins (e.g., CD46, CD55, and CD59) into the envelope of nascent virions. By acquiring host complement inhibitors, viruses prevent the deposition of complement components on their surface, thus resisting neutralization by complement or opsonization by phagocytic cells [24].

Interactions of human and animal complement systems with numerous viruses have been reported. This review will primarily focus on HCMV, and other members of the human herpesvirus family. For additional information, please refer to several recent publications on the interactions of the complement system with other viruses and viral complement-evasion strategies [19,20,21,22,23,24,25,26,27,28].

## 3. Interactions between Human Herpesviruses and the Complement System 

The human herpesvirus family includes herpes simplex virus 1 (HSV-1), herpes simplex virus 2 (HSV-2), varicella-zoster virus (VZV), Epstein–Barr virus (EBV), cytomegalovirus (HCMV), human herpesvirus 6A (HHV-6A), human herpesvirus 6B (HHV-6B), human herpesvirus 7 (HHV-7), and Kaposi’s sarcoma-associated herpesvirus (KSHV) [30]. Nearly all human herpesviruses have been documented to interact with the complement system to varying degrees and have collectively evolved unique mechanisms to exploit the complement system, including the following: (A) encoding viral complement inhibiting proteins, (B) using cellular complement receptors for viral entry into cells, and (C) incorporating host complement proteins on the surface of virions.

### 3.1. Virally Encoded Complement Inhibiting Proteins 

Glycoprotein C (gC) of HSV-1 was the first virally encoded molecule discovered to have complement-inhibiting functions [31]. Since then, complement-inhibiting proteins have been identified in HSV-1, HSV-2, and KSHV, and the details of their molecular inhibition of complement have been elucidated. HSV-1 and HSV-2 both encode gC, which binds to the central complement component 3 (C3) and its activation products (C3b, iC3b) [32]. gC also interferes with Factor H, Properdin, and C5, resulting in the further inhibition of complement [33,34,35]. Additionally, both HSV-1 and VZV encode glycoproteins gE and gI, which together form a complex that functions as an IgG Fc-gamma receptor (FcɣR) that occludes the Fc domain of IgG and inhibits antibody-dependent complement-mediated neutralization in the classical pathway [36,37,38]. Similarly, KSHV encodes the KSHV complement control protein (KCP/ORF4), a viral complement control protein homolog of human CD55 that decays the C3 convertase and inactivates C3b and C4 to inhibit complement [39,40]. Lastly, some evidence suggests the existence of a complement-regulating protein in EBV based on the following observations that (a) the incubation of purified EBV with human immune serum results in the cleavage of C3 into iC3, (b) EBV functions as a co-factor for Factor-I-mediated cleavage of C3b/iC3b and C4b/iC4b in a Factor-I-dependent mechanism, and (c) EBV accelerates the decay of the alternative, but not the classical C3 convertase [41]. Currently, no EBV proteins have been reported to have complement-regulating activities, nor are there any viral proteins encoded by EBV with significant homology to known complement regulatory proteins. Thus, in the absence of an EBV-encoded complement-regulating protein, one possible explanation for the ability of EBV to regulate complement is via the upregulation and incorporation of host membrane-bound complement-regulating proteins into the EBV viral envelope—a mechanism that has been previously observed in other human herpesviruses [42,43]. Taken together, future studies are needed to elucidate the precise molecular mechanism(s) employed by EBV to regulate complement activity.

### 3.2. Complement Proteins as Receptors for Viral Entry

An alternative strategy employed by herpesviruses to exploit the complement system involves using host membrane-bound complement proteins as receptors for viral entry. For example, HHV-6A encodes a protein complex composed of gH-gL-gQ, which serves as a viral ligand for CD46 (a membrane-bound complement inhibitor), an interaction that is necessary for HHV-6A infectivity and membrane fusion [44,45,46]. Similarly, EBV encodes gp350/220, which serves as a ligand for complement receptor 1 (CR1/CD35) [47] and complement receptor 2 (CR2/CD21) [48], permitting viral entry into B-cells.

### 3.3. Incorporation of Host Complement-Inhibiting Proteins on the Surface of Virions

In the absence of, or in addition to viral encoded complement-inhibiting proteins, some human herpesviruses incorporate host complement proteins on the viral envelope to prevent complement-mediated neutralization. For example, both HSV-1 and VZV incorporate host cellular CD59 (a membrane-bound host complement inhibitor) into the viral envelopes of nascent virions during infection [42,43], though in the case of VZV, this process was found to be tissue-specific. Nevertheless, the incorporation of host CD59 into the viral envelope would be expected to confer resistance of virions to complement-mediated neutralization owing to the function of CD59 in preventing the formation of the membrane attack complex on the membrane of host cells. Similarly, HSV-1 has also been reported to incorporate host CD55 (another membrane-bound host complement inhibitor) into the viral envelope during infection of human neuronal and embryonal skin cells [49]. Interestingly, HSV-1 infected neuronal cells had greater complement resistance compared to embryonal skin cells [49]—an observation that underscores the need to study the complement evasion mechanisms of herpesviruses in diverse cell types.

## 4. Associations and Reported Interactions between HCMV and Human Complement Proteins

The role of the complement system in infection and immunity to HCMV remains poorly understood even though the protective role of complement, and complement evasion mechanisms, have been characterized in detail for other human herpesviruses [24]. Herein, we describe how HCMV evades complement-mediated neutralization and highlight emerging and unorthodox roles of complement proteins in the HCMV viral lifecycle. A summary of associations and reported interactions between HCMV viral proteins and human complement proteins are listed in Table 2. 

### 4.1. Complement Evasion Using CD55, CD46, and CD59

The observation that complement has a negligible effect on HCMV virions in the absence of anti-HCMV antibodies gave rise to the hypothesis that HCMV may have evolved a mechanism to evade complement-mediated neutralization. However, initial studies of the HCMV genome revealed no viral proteins with significant homology to known complement-regulating proteins. Spiller et al. demonstrated that HCMV virions incubated in human serum (a source of complement) consumed complement activity and resulted in the deposition of activated early complement protein fragments (C3a, C3b) with the negligible deposition of terminal complement components (C9) and no loss of viral infectivity [50]. This observation implied that HCMV virions not only activate one or more pathways of complement but are able to interfere with steps in complement activation that precede the membrane attack complex [50,51]. Additional studies have demonstrated that HCMV evades complement-mediated neutralization by upregulating the membrane-bound host complement inhibitors CD55, CD46, and CD59 on infected cells and incorporating these proteins into the viral envelope of nascent HCMV virions [52].

The upregulation of CD55, CD46, and CD59 during HCMV infection in cells is notable, given that all three host proteins serve unique functions in regulating complement at various stages of the complement cascade and in multiple pathways. CD55, for instance, can inhibit all three pathways of complement by preventing both the assembly and accelerating the decay of C3 and C5 convertases [59]. Similarly, CD46 acts as a co-factor for the Factor-I-mediated inactivation of C3b and C4b, while CD59 prevents terminal MAC formation for all three complement pathways. While CD55, CD46, and CD59 all concomitantly serve important complement-inhibiting functions, CD55 is critically required for complement resistance based on the observation that HCMV virions incubated with anti-CD55 antibodies (but not anti-CD59 antibodies) are susceptible to complement-mediated neutralization [52].

Although incorporation of host CD55, CD46, and CD59 into the viral envelope of HCMV may mechanistically explain how HCMV virions resist complement-mediated neutralization, future studies are needed to elucidate whether this mechanism occurs passively or is actively mediated by viral proteins. Furthermore, given the broad cell tropism of HCMV and the ability of different cells to produce some or all complement proteins, future studies should investigate whether the viral acquisition of host membrane-bound complement proteins confers HCMV complement resistance among diverse HCMV-permissive cell types.

### 4.2. Viral Entry Regulated by CD46 and Mannose-Binding Lectin

Aside from contributing to HCMV complement resistance, emerging studies have described a role for host complement proteins in HCMV viral entry. In a recent study by Parsons et al., the authors identified an extracellular domain of host CD46 that functions as an important co-factor for HCMV infection in non-fibroblast cells (epithelial, trophoblasts, and endothelial cells) mediated by the viral pentameric complex gH-gL-UL128-UL130-UL131a [60]. These findings agree with additional studies reporting positive protein–protein interactions between CD46 and components of the HCMV pentamer complex in vitro [58,61].

While some complement proteins such as CD46 may facilitate HCMV infection, other studies have reported a possible protective role of other complement proteins such as MBL in inhibiting HCMV infection. Mannose-binding lectin (MBL) is a major protein released during the acute phase response to infection, acting as a pattern recognition molecule for foreign polysaccharides such as N-acetylglucosamine (found in diverse microorganisms, including viruses) and opsonins in the lectin pathway of complement [62]. MBL contributes to the neutralization of many highly pathogenic human viruses by activating the lectin pathway of complement and by enhancing opsonization of viruses by phagocytic cells [63]. Despite the absence of a clear molecular mechanism for the role of MBL in HCMV infection, several studies have implicated MBL as having a protective role in immunity to HCMV. For example, individuals with genetic deficiencies in MBL or polymorphisms that result in low MBL expression have an increased susceptibility to viral infections, including HCMV [64,65]. Similarly, a previous study by Wu et al. reported that the exogenous addition of either serum-purified or recombinant MBL can inhibit HCMV infection in human embryonic pulmonary fibroblasts in vitro, and the inhibition could be reduced proportionally with increasing concentrations of mannan, a major ligand recognized by MBL [57]. Although no specific mechanism was put forward, the preceding observations suggest that MBL recognizes an HCMV viral protein that is surface-exposed and may be involved in the initial steps of viral entry. The entry of HCMV into fibroblasts depends on envelope glycoprotein complexes composed of gM/gN, gB, or gH/gL/gO [66]—of which, gB, gH, and gO have predicted or reported N-linked glycosylation sites that would be expected to be recognized by MBL [67].

Taken together, it is possible that MBL may recognize glycosylated residues present on surface-exposed envelope glycoproteins involved in HCMV viral entry and may sterically hinder these molecules from engaging with host cell receptors, thereby inhibiting viral entry. Moreover, this hypothesis may mechanistically explain the conclusions of studies that report a strong association between low MBL serum levels and an increased risk of HCMV infection in certain populations [64]. Future studies are needed to identify possible HCMV-encoded MBL ligands and elucidate the molecular mechanisms responsible for MBL-mediated inhibition in HCMV infection.

### 4.3. Effect of C1qBP on Viral DNA Replication and Gene Expression

In the complement system, C1q-binding protein (also referred to as: C1qBP, gC1qR, HABP1, and p32) serve as a receptor for C1q, allowing C1qBP to regulate the classical pathway of complement by inhibiting C1 activation through engagement with C1q [68]. However, C1qBP is a multifunctional protein found extracellularly and in many subcellular locations including the cell membrane, mitochondria, cytoplasm, and nucleus [69], where it interacts with multiple host ligands to regulate diverse cellular processes, including maintaining mitochondrial homeostasis and oxidative phosphorylation [70], mediating nucleus–mitochondrial interactions [71], mitochondrial antiviral signaling (MAVS) [72], and even transcriptional regulation [73].

Multiple studies have demonstrated that HCMV infection in cells results in an increased expression of C1qBP [74,75] and the translocation of C1qBP from the cytoplasm to the nucleus via an unknown mechanism [76]. Once in the nucleus, C1qBP co-localizes with UL84 and UL44 and is reported to directly interact with both proteins by mass-spectrometry and co-immunoprecipitation [56,76]. These results are significant given that UL84 is a major regulator of IE2/IE86, a nuclear phosphoprotein and transcription factor that drives expression of immediate-early IE genes necessary to prime infected cells for HCMV viral replication [77]. Additionally, UL84 serves as a bridge between the complex network of proteins involved in HCMV gene expression and viral DNA replication (UL44, UL54, UL57, UL70, UL102, UL105) [78]. Taken together, the interactions between C1qBP-UL84 and C1qBP-UL44 strongly suggest that C1qBP may be involved in both HCMV gene expression and viral DNA replication.

C1qBP is thought to be involved in gene expression and RNA splicing due to its robust transcription-activation domain and association with host transcription factors like forkhead box C1 (FOXC-1) [79], as well as pre-mRNA splicing factor 2 (SF2) [80]. While unconventional, C1qBP’s involvement in transcriptional regulation extends to related human herpesviruses. Notably, in Epstein–Barr virus (EBV), C1qBP regulates the Epstein–Barr Nuclear Antigen-1 (EBNA-1) transcription necessary for maintaining the viral chromosome during latency and facilitating EBV latent cell cycle DNA replication [81,82].

Taken together, the importance of C1qBP in one or more steps of the HCMV viral lifecycle is highlighted by the fact that the reduced expression of C1qBP during HCMV infection ablates the production of essential viral proteins and significantly inhibits viral replication [53]. In contrast, the concomitant over-expression of C1qBP during HCMV infection increases viral titers and results in the greater release of viral particles [55], suggesting that C1qBP serves an essential role in HCMV replication—though the full repertoire of C1qBP-interacting viral proteins encoded by HCMV remains unknown.

### 4.4. Role of C1qBP in Nuclear Egress

C1qBP has been reported to be involved in the nuclear egress of several human herpesviruses including HSV-1 [83] and possibly EBV [84], though it has been extensively characterized in HCMV, serving as a major adaptor protein hub that functions in coordination with viral and other host proteins to form the nuclear egress complex (NEC). The HCMV NEC is a multiprotein complex composed of viral proteins (UL50, UL53, UL97) and host cell proteins including but not limited to C1qBP, Lamin B receptor (LBR), and Protein Kinase C (PKC) [53]. This egress process requires the phosphorylation of C1qBP by the UL97 viral kinase as well as the phosphorylation of UL50 and other proteins in the nuclear lamina [55,85,86].

Previous studies have documented direct protein–protein interactions between C1qBP and key HCMV proteins compromising the NEC, including C1qBP-UL97 [55], C1qBP-UL50 [53], and C1qBP-UL53 [53,54]. However, conflicting findings exist regarding the C1qBP-UL53 interaction, with some studies reporting no interaction in yeast two-hybrid screens [54], a weak interaction in co-immunoprecipitation assays [87], and a positive interaction in mass spectrometry analyses [53]. These disparities highlight the challenge in elucidating the viral and host composition of the HCMV NEC, which likely includes numerous unidentified viral and host proteins. Additionally, high-throughput proteomic studies may bias stronger protein–protein interactions or require certain enrichment thresholds, and therefore overlook weaker and less abundant, yet biologically significant protein–protein interactions. Thus, future research is warranted to unravel the complete repertoire of HCMV-encoded proteins that interact with C1qBP during nuclear egress.

## 5. Future Directions

HCMV, as a member of the human herpesvirus family, has evolved an array of sophisticated strategies to exploit and evade various components of both innate and adaptive immune responses. However, few studies have focused on the interactions of HCMV with the complement system compared to components of the other immune responses. Understanding the nature of complement interactions with HCMV and other herpesviruses and the mechanisms of their complement evasion not only provides insights into viral pathogenesis but also has the potential to inform the development of novel antiviral agents and vaccines. For example, the targeted inhibition of HCMV viral proteins that mediate complement evasion mechanisms may render HCMV virions or infected cells susceptible to neutralization by complement alone or in conjunction with other immune factors such as antibodies and cells, which may overall bolster host immunity.

Considering the known strategy of upregulating host complement-inhibiting proteins during HCMV infection, and the absence of reported HCMV-encoded ligands that directly inhibit complement, it is possible that HCMV may have evolved discrete mechanisms to resist complement indirectly rather than directly. For example, HCMV encodes viral Fc-gamma receptors including gp34 (TRL11/IRL11) and gp68 (UL119-UL118) [88,89], both of which bind all four human IgG subclasses (IgG1, IgG2, IgG3, and IgG4) [90]. However, unlike viral Fc-gamma receptor homologs in other herpesviruses, the functional repertoire of HCMV-encoded Fc-gamma receptor homologs remains incompletely understood [91]. Since the engagement of C1q to the Fc region of antibodies initiates the activation of the classical pathway of complement, occluding antibody Fc regions by viral Fc-gamma-receptors may represent an additional strategy employed by HCMV to evade complement via an antibody-dependent mechanism (i.e., antibody-dependent complement-mediated neutralization). Importantly, the salient contribution of complement in antibody neutralization is underscored by the fact that some anti-HCMV monoclonal antibodies or antibodies induced by experimental vaccination can be partially, or even completely complement-dependent, and can vary by HCMV antigen and protein conformation [92,93,94].

Similarly, the ability of complement to shape adaptive immunity also has significant implications for the development of novel adjuvants and vaccines for HCMV and other human herpesviruses. In a pivotal study by Da Costa et al., the authors demonstrated the critical role of complement in the humoral response to HSV-1 infection. Mice deficient in C3, C4, or CD21/CD35 had significantly lower IgG antibody responses and germinal centers compared to wild-type controls following infection with HSV-1 [95]. These results reveal an important role for complement not only as a first line of innate immunity, but also in the regulation of adaptive memory B-cell responses to viral-infected cell antigens in both blood and peripheral tissues. In this context, the inhibition of complement by human herpesviruses may serve the following dual functions: a) conferring protection to virions from complement-mediated neutralization and b) suppressing the induction and maintenance of robust IgG antibody and memory responses. Thus, continued research into the intricate interactions between herpesviruses and the complement system holds the promise of unveiling new therapeutic modalities for combating herpesvirus infections and bolstering host immunity.

Traditionally, complement proteins are viewed as innate immune components with extracellular functions. However, recent studies have revealed their diverse and ever-expanding intracellular roles. In its intracellular state, the complement system (referred to as the ‘complosome’) is reported to participate in various cellular processes, including maintaining T-cell homeostasis and differentiation [96], regulating apoptosis [97], regulating cell metabolism [98], and directly neutralizing intracellular pathogens such as viruses [99]. In a groundbreaking study by Tam et al., the authors identified an intracellular C3 sensing mechanism, whereby the recognition of C3b-coated pathogens in the cytosol led to the activation of mitochondrial antiviral signaling (MAVS) pathways, inflammatory cytokine synthesis, and pathogen degradation via the proteasomal pathway [99]. Remarkably, this mechanism appears to be highly conserved based on the observation that C3 from various mammalian species could induce this newly discovered cell-autonomous immunity, independent of specific PAMPs [99]. Conceivably, viruses that encode complement-inhibiting proteins or that co-opt host complement-inhibiting proteins to limit C3b deposition on their surface would be at a great advantage in evading this intracellular C3-sensing mechanism.

Given the fact that HCMV encodes an array of factors modulating many branches of both the innate and adaptive immune responses, it is reasonable to suggest that HCMV may employ unique and novel mechanisms to counteract all three complement pathways by encoding numerous factors to interact with different host proteins involved in each pathway. Further studies on HCMV interactions with the complement system will provide insight into our understanding of HCMV infection and pathogenesis. Moreover, these studies will facilitate the development of novel anti-HCMV compounds and new HCMV vaccines for the treatment and prevention of HCMV infection and associated diseases.

## Figures and Tables

**Table 1:** Major proteins of the complement system and their functions. The complement system comprises a diverse array of proteins that upon activation orchestrate a cascade of proteolytic reactions that result in the elimination of microorganisms. Complement proteins can be classified into distinct protein classes that include pattern recognition molecules, activating enzymes, opsonins, inflammatory mediators, complement regulators, complement receptors, and terminal complement components that form the membrane attack complex (MAC). Table lists a summary of proteins that make up the complement system, their functions, and their involvement in different pathways as described previously [5,6,7,8,9,10,11,12,13,14].

Class	Name	Function	Pathway
Pattern Recognition Molecules	C1q	C1q recognizes antibody or antibody–antigen complexes and contributes to the activation of the classical pathway via the C1 complex (C1rC1s)^2^.	Classical
	Mannose-Binding Lectin (MBL)	MBL recognizes foreign carbohydrates on microbial surfaces and contributes to the activation of the lectin pathway mediated by MASPs.	Lectin
	Ficolins	Pattern recognition of foreign carbohydrates, same as above.	Lectin
	Collectins	Pattern recognition of foreign carbohydrates, same as above.	Lectin
Activating Enzymes	C1r	C1r is a serine protease and subcomponent of the C1 complex. Upon binding to C1q, C1r proteolytically cleaves C1s to initiate classical pathway activation.	Classical
	C1s	C1s is a serine protease and subcomponent of the C1 complex. Upon activation by C1r, C1s proteolytically cleaves C4 (into C4a, C4b) and C2 (into C2a, C2b) in the classical pathway.	Classical
	C2a	C2a assembles with C4b to form the C3 convertase (C4bC2a) of the classical and lectin pathways.	Classical, Lectin
	Factor D (FD)	FD is a serine protease that cleaves FB to produce Bb in the alternative pathway.	Alternative
	Factor B (FB)	FB is a serine protease that provides catalytic activity and is a subcomponent of the C3 convertase (C3bBb) and C5 convertase (C3bBbC3b) of the alternative pathway.	Alternative
	MBL-Associated Serine Protease 1 (MASP-1)	MASP-1 is a serine protease that cleaves C2 (into C2a and C2b) and activates MASP-2 in the lectin pathway.	Lectin
	MBL-Associated Serine Protease 2 (MASP-2)	MASP-2 is a serine protease that cleaves C4 (into C4a and C4b) and C2 (into C2a and C2b) in conjunction with MASP-1 in the lectin pathway.	Lectin
Opsonins	C4b	C4b binds covalently to complement activating surfaces and is an opsonin recognized by complement receptors on cells. C4b is also a subcomponent of the C3 convertase (C4bC2a) and C5 convertase (C4bC2aC3b) of the classical and lectin pathways.	Classical, Lectin
	C3b	C3b is deposited on complement activating surfaces and is a potent opsonin recognized by complement receptors on cells. C3b is also a subcomponent of the C3 convertase (C3bBb) and C5 convertase (C3bBbC3b) of the alternative pathway, as well as the C5 convertase (C4bC2aC3b) of the classical and lectin pathways.	All
Inflammatory Mediators	C3a	C3a is a potent anaphylatoxin and is recognized by C3a receptors on cells. C3a can induce inflammation, extravasation, and chemotaxis of cells to sites of infection.	All
	C5a	C5a is a potent anaphylatoxin and is recognized by C5a receptors on cells. C5a can induce inflammation, extravasation, and chemotaxis of cells to sites of infection.	All
Membrane Attack Complex (MAC)	C5b	The MAC is a multiprotein complex assembled during the terminal stage of the complement cascade that produces a pore on complement-activating surfaces, resulting in membrane lysis.	All
	C6		All
	C7		All
	C8		All
	C9		All
Complement Regulators	C1 Esterase Inhibitor (C1-INH)	C1-INH is a protease inhibitor that regulates the classical pathway of complement by inhibiting C1r and C1s serine proteases in the C1 complex. C1-INH also regulates the lectin pathway by inhibiting the cleavage of C4 and C2 by MASPs.	Classical, Lectin
	C1q-Binding Protein (C1qBP/gC1qBP/p32)	C1qBP regulates the classical pathway by binding to C1q and inhibiting the activation of the C1 complex.	Classical
	C4b-Binding Protein (C4bp)	C4bp regulates the classical and lectin pathways by binding to C4b and preventing the formation, or accelerating the decay, of the C3 convertase (C4bC2a) in both pathways.	Classical, Lectin
	Complement Receptor 1 (CR1/CD35)	CD35 regulates the classical, lectin, and alternative pathways by serving as a receptor for C3b and C4b. CD35 accelerates the decay of the classical/lectin C3 convertase (C4bC2a) and C5 convertase (C4bC2aC3b). Similarly, CD35 can accelerate the decay of the alternative pathway C3 convertase (C3bBb) and C5 convertase (C3bBbC3b).	All
	Membrane Cofactor Protein (MCP/CD46)	CD46 regulates the classical, lectin, and alternative pathways by serving as a co-factor for the inactivation of C3b and C4b by Factor I (FI).	All
	Decay-Accelerating Factor (DAF/CD55)	CD55 regulates the classical, lectin, and alternative pathways by binding to C4b and C3b to inhibit the assembly, or accelerate the decay, of C3 convertases (C4bC2a and C3bBb) and C5 convertases (C4bC2aC3b and C3bBbC3b) in all three pathways.	All
	Factor H (FH)	FH regulates the alternative pathway by inhibiting the assembly of the alternative pathway C3 convertase (C3bBb) and C5 convertase (C3bBbC3b) by competing with Factor B (FB) for the binding of C3b. FH also facilitates the decay of the C3 and C5 convertases by displacing Bb and acting as a co-factor for the Factor-I-mediated cleavage and inactivation of C3b.	Alternative
	Factor I (FI)	FI regulates the classical, lectin, and alternative pathways by cleaving and degrading C3b and C4b in the presence of co-factors such as Factor H (FH), C4b-binding protein (C4bp), CR1 (CD35), or MCP (CD46).	All
	Properdin (P)	Properdin positively regulates and enhances complement by stabilizing the alternative pathway C3 convertase (C3bBb).	Alternative
	MAC- inhibitory protein (MAC-IP/CD59)	CD59 regulates the classical, lectin, and alternative pathways by inhibiting the polymerization of C9 to prevent the assembly of the MAC (C5bC6C7C8C9).	All
Complement Receptors	Complement Receptor 1 (CR1/CD35)	CD35 recognizes C3b and C4b on complement-activating surfaces and enhances phagocytosis. CD35 also has complement-regulating functions, as described above.	
	Complement Receptor 2 (CR2/CD21)	CD21 binds to C3d-coated antigens and can regulate B-cell activation and antigen processing and presentation.	
	Complement Receptor 3 (CR3/CD11b-CD18)	CD11b/CD18 recognizes iC3b and facilitates phagocytosis, adhesion, and immune cell trafficking to sites of infection.	
	Complement Receptor 4 (CR4/CD11c-CD18)	CD11c/CD18 recognizes iC3b and facilitates phagocytosis, adhesion, and immune cell trafficking to sites of infection. CD11c/CD18 may also contribute to antigen presentation.	

**Table 2:** Associations and reported interactions between HCMV and human complement proteins.

Complement Protein	HCMV Protein(s)	Biological Process	Reference
CD55	Unknown	Complement evasion	[50,51,52]
CD59	Unknown	Complement evasion	[50,51,52]
C1qBP	UL50	Nuclear egress complex (NEC)	[53]
	UL53		[53,54]
	UL97		[55]
	UL84	Viral gene expression	[56]
MBL	Unknown	Viral entry (Pulmonary Fibroblasts)	[57]
CD46	gH/gL/UL128-130-131A	Viral entry (Epithelial, Trophoblasts)	[50,58]

## Data Availability

Not applicable.

## References

[1] Al-Sharif W.Z., Sunyer J.O., Lambris J.D., Smith L.C. (1998). Sea urchin coelomocytes specifically express a homologue of the complement component C3. J. Immunol..

[2] Levashina E.A., Moita L.F., Blandin S., Vriend G., Lagueux M., Kafatos F.C. (2001). Conserved role of a complement-like protein in phagocytosis revealed by dsRNA knockout in cultured cells of the mosquito, Anopheles gambiae. Cell.

[3] Kimura A., Sakaguchi E., Nonaka M. (2009). Multi-component complement system of Cnidaria: C3, Bf, and MASP genes expressed in the endodermal tissues of a sea anemone, Nematostella vectensis. Immunobiology.

[4] Lubbers R., van Essen M.F., van Kooten C., Trouw L.A. (2017). Production of complement components by cells of the immune system. Clin. Exp. Immunol..

[5] Merle N.S., Church S.E., Fremeaux-Bacchi V., Roumenina L.T. (2015). Complement System Part I—Molecular Mechanisms of Activation and Regulation. Front. Immunol..

[6] Mortensen S.A., Sander B., Jensen R.K., Pedersen J.S., Golas M.M., Jensenius J.C., Hansen A.G., Thiel S., Andersen G.R. (2017). Structure and activation of C1, the complex initiating the classical pathway of the complement cascade. Proc. Natl. Acad. Sci. USA.

[7] Pangburn M.K., Rawal N. (2002). Structure and function of complement C5 convertase enzymes. Biochem. Soc. Trans..

[8] Heja D., Kocsis A., Dobo J., Szilagyi K., Szasz R., Zavodszky P., Pal G., Gal P. (2012). Revised mechanism of complement lectin-pathway activation revealing the role of serine protease MASP-1 as the exclusive activator of MASP-2. Proc. Natl. Acad. Sci. USA.

[9] Podack E.R., Tschoop J., Muller-Eberhard H.J. (1982). Molecular organization of C9 within the membrane attack complex of complement. Induction of circular C9 polymerization by the C5b-8 assembly. J. Exp. Med..

[10] Morgan B.P. (1999). Regulation of the complement membrane attack pathway. Crit. Rev. Immunol..

[11] Santos-Lopez J., de la Paz K., Fernandez F.J., Vega M.C. (2023). Structural biology of complement receptors. Front. Immunol..

[12] Dunkelberger J.R., Song W.C. (2010). Complement and its role in innate and adaptive immune responses. Cell Res..

[13] Klos A., Tenner A.J., Johswich K.O., Ager R.R., Reis E.S., Kohl J. (2009). The role of the anaphylatoxins in health and disease. Mol. Immunol..

[14] Markiewski M.M., Lambris J.D. (2007). The role of complement in inflammatory diseases from behind the scenes into the spotlight. Am. J. Pathol..

[15] Xie C.B., Jane-Wit D., Pober J.S. (2020). Complement Membrane Attack Complex: New Roles, Mechanisms of Action, and Therapeutic Targets. Am. J. Pathol..

[16] Killick J., Morisse G., Sieger D., Astier A.L. (2018). Complement as a regulator of adaptive immunity. Semin. Immunopathol..

[17] West E.E., Kolev M., Kemper C. (2018). Complement and the Regulation of T Cell Responses. Annu. Rev. Immunol..

[18] Clarke E.V., Tenner A.J. (2014). Complement modulation of T cell immune responses during homeostasis and disease. J. Leukoc. Biol..

[19] Murugaiah V., Varghese P.M., Beirag N., De Cordova S., Sim R.B., Kishore U. (2021). Complement Proteins as Soluble Pattern Recognition Receptors for Pathogenic Viruses. Viruses.

[20] Sinha A., Singh A.K., Kadni T.S., Mullick J., Sahu A. (2021). Virus-Encoded Complement Regulators: Current Status. Viruses.

[21] Mellors J., Tipton T., Longet S., Carroll M. (2020). Viral Evasion of the Complement System and Its Importance for Vaccines and Therapeutics. Front. Immunol..

[22] Agrawal P., Sharma S., Pal P., Ojha H., Mullick J., Sahu A. (2020). The imitation game: A viral strategy to subvert the complement system. FEBS Lett..

[23] Maloney B.E., Perera K.D., Saunders D.R.D., Shadipeni N., Fleming S.D. (2020). Interactions of viruses and the humoral innate immune response. Clin. Immunol..

[24] Agrawal P., Nawadkar R., Ojha H., Kumar J., Sahu A. (2017). Complement Evasion Strategies of Viruses: An Overview. Front. Microbiol..

[25] Ojha H., Panwar H.S., Gorham R.D., Morikis D., Sahu A. (2014). Viral regulators of complement activation: Structure, function and evolution. Mol. Immunol..

[26] Heggi M.T., Nour El-Din H.T., Morsy D.I., Abdelaziz N.I., Attia A.S. (2023). Microbial evasion of the complement system: A continuous and evolving story. Front. Immunol..

[27] Stoermer K.A., Morrison T.E. (2011). Complement and viral pathogenesis. Virology.

[28] Varkoly K., Beladi R., Hamada M., McFadden G., Irving J., Lucas A.R. (2023). Viral SERPINS-A Family of Highly Potent Immune-Modulating Therapeutic Proteins. Biomolecules.

[29] Miller C.G., Shchelkunov S.N., Kotwal G.J. (1997). The cowpox virus-encoded homolog of the vaccinia virus complement control protein is an inflammation modulatory protein. Virology.

[30] Arvin A., Campadelli-Fiume G., Mocarski E., Moore P.S., Roizman B., Whitley R., Yamanishi K. Human Herpesviruses: Biology, Therapy, and Immunoprophylaxis, Cambridge University Press: Cambridge, UK, 2007.

[31] Friedman H.M., Glorioso J.C., Cohen G.H., Hastings J.C., Harris S.L., Eisenberg R.J. (1986). Binding of complement component C3b to glycoprotein gC of herpes simplex virus type 1: Mapping of gC-binding sites and demonstration of conserved C3b binding in low-passage clinical isolates. J. Virol..

[32] Fries L.F., Friedman H.M., Cohen G.H., Eisenberg R.J., Hammer C.H., Frank M.M. (1986). Glycoprotein C of herpes simplex virus 1 is an inhibitor of the complement cascade. J. Immunol..

[33] Harris S.L., Frank I., Yee A., Cohen G.H., Eisenberg R.J., Friedman H.M. (1990). Glycoprotein C of herpes simplex virus type 1 prevents complement-mediated cell lysis and virus neutralization. J. Infect. Dis..

[34] Huemer H.P., Wang Y., Garred P., Koistinen V., Oppermann S. (1993). Herpes simplex virus glycoprotein C: Molecular mimicry of complement regulatory proteins by a viral protein. Immunology.

[35] Kostavasili I., Sahu A., Friedman H.M., Eisenberg R.J., Cohen G.H., Lambris J.D. (1997). Mechanism of complement inactivation by glycoprotein C of herpes simplex virus. J. Immunol..

[36] Frank I., Friedman H.M. (1989). A novel function of the herpes simplex virus type 1 Fc receptor: Participation in bipolar bridging of antiviral immunoglobulin G. J. Virol..

[37] Litwin V., Grose C. (1992). Herpesviral Fc receptors and their relationship to the human Fc receptors. Immunol. Res..

[38] Litwin V., Jackson W., Grose C. (1992). Receptor properties of two varicella-zoster virus glycoproteins, gpI and gpIV, homologous to herpes simplex virus gE and gI. J. Virol..

[39] Spiller O.B., Blackbourn D.J., Mark L., Proctor D.G., Blom A.M. (2003). Functional activity of the complement regulator encoded by Kaposi’s sarcoma-associated herpesvirus. J. Biol. Chem..

[40] Spiller O.B., Mark L., Blue C.E., Proctor D.G., Aitken J.A., Blom A.M., Blackbourn D.J. (2006). Dissecting the regions of virion-associated Kaposi’s sarcoma-associated herpesvirus complement control protein required for complement regulation and cell binding. J. Virol..

[41] Mold C., Bradt B.M., Nemerow G.R., Cooper N.R. (1988). Epstein-Barr virus regulates activation and processing of the third component of complement. J. Exp. Med..

[42] Stegen C., Yakova Y., Henaff D., Nadjar J., Duron J., Lippe R. (2013). Analysis of virion-incorporated host proteins required for herpes simplex virus type 1 infection through a RNA interference screen. PLoS ONE.

[43] Wang W., Wang X., Yang L., Fu W., Pan D., Liu J., Ye J., Zhao Q., Zhu H., Cheng T. (2016). Modulation of host CD59 expression by varicella-zoster virus in human xenografts in vivo. Virology.

[44] Santoro F., Kennedy P.E., Locatelli G., Malnati M.S., Berger E.A., Lusso P. (1999). CD46 is a cellular receptor for human herpesvirus 6. Cell.

[45] Mori Y., Yang X., Akkapaiboon P., Okuno T., Yamanishi K. (2003). Human herpesvirus 6 variant A glycoprotein H-glycoprotein L-glycoprotein Q complex associates with human CD46. J. Virol..

[46] Mori Y., Seya T., Huang H.L., Akkapaiboon P., Dhepakson P., Yamanishi K. (2002). Human herpesvirus 6 variant A but not variant B induces fusion from without in a variety of human cells through a human herpesvirus 6 entry receptor, CD46. J. Virol..

[47] Ogembo J.G., Kannan L., Ghiran I., Nicholson-Weller A., Finberg R.W., Tsokos G.C., Fingeroth J.D. (2013). Human complement receptor type 1/CD35 is an Epstein-Barr Virus receptor. Cell Rep..

[48] Tanner J., Weis J., Fearon D., Whang Y., Kieff E. (1987). Epstein-Barr virus gp350/220 binding to the B lymphocyte C3d receptor mediates adsorption, capping, and endocytosis. Cell.

[49] Rautemaa R., Helander T., Meri S. (2002). Herpes simplex virus 1 infected neuronal and skin cells differ in their susceptibility to complement attack. Immunology.

[50] Spiller O.B., Morgan B.P., Tufaro F., Devine D.V. (1996). Altered expression of host-encoded complement regulators on human cytomegalovirus-infected cells. Eur. J. Immunol..

[51] Spiller O.B., Hanna S.M., Devine D.V., Tufaro F. (1997). Neutralization of cytomegalovirus virions: The role of complement. J. Infect. Dis..

[52] Spear G.T., Lurain N.S., Parker C.J., Ghassemi M., Payne G.H., Saifuddin M. (1995). Host cell-derived complement control proteins CD55 and CD59 are incorporated into the virions of two unrelated enveloped viruses. Human T cell leukemia/lymphoma virus type I (HTLV-I) and human cytomegalovirus (HCMV). J. Immunol..

[53] Milbradt J., Kraut A., Hutterer C., Sonntag E., Schmeiser C., Ferro M., Wagner S., Lenac T., Claus C., Pinkert S. (2014). Proteomic analysis of the multimeric nuclear egress complex of human cytomegalovirus. Mol. Cell Proteom..

[54] Milbradt J., Auerochs S., Marschall M. (2007). Cytomegaloviral proteins pUL50 and pUL53 are associated with the nuclear lamina and interact with cellular protein kinase C. J. Gen. Virol..

[55] Marschall M., Marzi A., aus dem Siepen P., Jochmann R., Kalmer M., Auerochs S., Lischka P., Leis M., Stamminger T. (2005). Cellular p32 recruits cytomegalovirus kinase pUL97 to redistribute the nuclear lamina. J. Biol. Chem..

[56] Gao Y., Colletti K., Pari G.S. (2008). Identification of human cytomegalovirus UL84 virus- and cell-encoded binding partners by using proteomics analysis. J. Virol..

[57] Wu W., Chen Y., Qiao H., Tao R., Gu W., Shang S. (2012). Human mannose-binding lectin inhibits human cytomegalovirus infection in human embryonic pulmonary fibroblast. APMIS.

[58] Stein K.R., Gardner T.J., Hernandez R.E., Kraus T.A., Duty J.A., Ubarretxena-Belandia I., Moran T.M., Tortorella D. (2019). CD46 facilitates entry and dissemination of human cytomegalovirus. Nat. Commun..

[59] Kim D.D., Song W.C. (2006). Membrane complement regulatory proteins. Clin. Immunol..

[60] Parsons A.J., Stein K.R., Atanasoff K.E., Ophir S.I., Casado J.P., Tortorella D. (2023). The CD46 ectodomain participates in human cytomegalovirus infection of epithelial cells. J. Gen. Virol..

[61] Martinez-Martin N., Marcandalli J., Huang C.S., Arthur C.P., Perotti M., Foglierini M., Ho H., Dosey A.M., Shriver S., Payandeh J. (2018). An Unbiased Screen for Human Cytomegalovirus Identifies Neuropilin-2 as a Central Viral Receptor. Cell.

[62] Kilpatrick D.C. (2003). Introduction to mannan-binding lectin. Biochem. Soc. Trans..

[63] Mason C.P., Tarr A.W. (2015). Human lectins and their roles in viral infections. Molecules.

[64] Hu Y., Wu D., Tao R., Shang S. (2010). Association between mannose-binding lectin gene polymorphism and pediatric cytomegalovirus infection. Viral Immunol..

[65] Cervera C., Lozano F., Linares L., Anton A., Balderramo D., Suarez B., Pascal M., Sanclemente G., Cofan F., Ricart M.J. (2009). Influence of mannose-binding lectin gene polymorphisms on the invasiveness of cytomegalovirus disease after solid organ transplantation. Transplant. Proc..

[66] Nguyen C.C., Kamil J.P. (2018). Pathogen at the Gates: Human Cytomegalovirus Entry and Cell Tropism. Viruses.

[67] Chen Y., Liu W., Luo B. (2023). The Effects of Herpes Virus Glycoprotein Glycosylation On Viral Infection and Pathogenesis. Future Virol..

[68] Pednekar L., Pathan A.A., Paudyal B., Tsolaki A.G., Kaur A., Abozaid S.M., Kouser L., Khan H.A., Peerschke E.I., Shamji M.H. (2016). Analysis of the Interaction between Globular Head Modules of Human C1q and Its Candidate Receptor gC1qR. Front. Immunol..

[69] van Leeuwen H.C., O’Hare P. (2001). Retargeting of the mitochondrial protein p32/gC1Qr to a cytoplasmic compartment and the cell surface. J. Cell Sci..

[70] Muta T., Kang D., Kitajima S., Fujiwara T., Hamasaki N. (1997). p32 protein, a splicing factor 2-associated protein, is localized in mitochondrial matrix and is functionally important in maintaining oxidative phosphorylation. J. Biol. Chem..

[71] Chen R., Xiao M., Gao H., Chen Y., Li Y., Liu Y., Zhang N. (2016). Identification of a novel mitochondrial interacting protein of C1QBP using subcellular fractionation coupled with CoIP-MS. Anal. Bioanal. Chem..

[72] Song K., Wu Y., Fu B., Wang L., Hao W., Hua F., Sun Y., Dorf M.E., Li S. (2021). Leaked Mitochondrial C1QBP Inhibits Activation of the DNA Sensor cGAS. J. Immunol..

[73] Yu L., Zhang Z., Loewenstein P.M., Desai K., Tang Q., Mao D., Symington J.S., Green M. (1995). Molecular cloning and characterization of a cellular protein that interacts with the human immunodeficiency virus type 1 Tat transactivator and encodes a strong transcriptional activation domain. J. Virol..

[74] Nightingale K., Lin K.M., Ravenhill B.J., Davies C., Nobre L., Fielding C.A., Ruckova E., Fletcher-Etherington A., Soday L., Nichols H. (2018). High-Definition Analysis of Host Protein Stability during Human Cytomegalovirus Infection Reveals Antiviral Factors and Viral Evasion Mechanisms. Cell Host Microbe.

[75] Nobre L.V., Nightingale K., Ravenhill B.J., Antrobus R., Soday L., Nichols J., Davies J.A., Seirafian S., Wang E.C., Davison A.J. (2019). Human cytomegalovirus interactome analysis identifies degradation hubs, domain associations and viral protein functions. eLife.

[76] Du G., Stinski M.F. (2013). Interaction network of proteins associated with human cytomegalovirus IE2-p86 protein during infection: A proteomic analysis. PLoS ONE.

[77] Marchini A., Liu H., Zhu H. (2001). Human cytomegalovirus with IE-2 (UL122) deleted fails to express early lytic genes. J. Virol..

[78] Pari G.S. (2008). Nuts and bolts of human cytomegalovirus lytic DNA replication. Curr. Top. Microbiol. Immunol..

[79] Huang L., Chi J., Berry F.B., Footz T.K., Sharp M.W., Walter M.A. (2008). Human p32 is a novel FOXC1-interacting protein that regulates FOXC1 transcriptional activity in ocular cells. Invest. Ophthalmol. Vis. Sci..

[80] Petersen-Mahrt S.K., Estmer C., Ohrmalm C., Matthews D.A., Russell W.C., Akusjarvi G. (1999). The splicing factor-associated protein, p32, regulates RNA splicing by inhibiting ASF/SF2 RNA binding and phosphorylation. EMBO J..

[81] Van Scoy S., Watakabe I., Krainer A.R., Hearing J. (2000). Human p32: A coactivator for Epstein-Barr virus nuclear antigen-1-mediated transcriptional activation and possible role in viral latent cycle DNA replication. Virology.

[82] Wang Y., Finan J.E., Middeldorp J.M., Hayward S.D. (1997). P32/TAP, a cellular protein that interacts with EBNA-1 of Epstein-Barr virus. Virology.

[83] Liu Z., Kato A., Oyama M., Kozuka-Hata H., Arii J., Kawaguchi Y. (2015). Role of Host Cell p32 in Herpes Simplex Virus 1 De-Envelopment during Viral Nuclear Egress. J. Virol..

[84] Changotra H., Turk S.M., Artigues A., Thakur N., Gore M., Muggeridge M.I., Hutt-Fletcher L.M. (2016). Epstein-Barr virus glycoprotein gM can interact with the cellular protein p32 and knockdown of p32 impairs virus. Virology.

[85] Sharma M., Bender B.J., Kamil J.P., Lye M.F., Pesola J.M., Reim N.I., Hogle J.M., Coen D.M. (2015). Human cytomegalovirus UL97 phosphorylates the viral nuclear egress complex. J. Virol..

[86] Sharma M., Kamil J.P., Coughlin M., Reim N.I., Coen D.M. (2014). Human cytomegalovirus UL50 and UL53 recruit viral protein kinase UL97, not protein kinase C, for disruption of nuclear lamina and nuclear egress in infected cells. J. Virol..

[87] Milbradt J., Auerochs S., Sticht H., Marschall M. (2009). Cytomegaloviral proteins that associate with the nuclear lamina: Components of a postulated nuclear egress complex. J. Gen. Virol..

[88] Atalay R., Zimmermann A., Wagner M., Borst E., Benz C., Messerle M., Hengel H. (2002). Identification and expression of human cytomegalovirus transcription units coding for two distinct Fcgamma receptor homologs. J. Virol..

[89] Lilley B.N., Ploegh H.L., Tirabassi R.S. (2001). Human cytomegalovirus open reading frame TRL11/IRL11 encodes an immunoglobulin G Fc-binding protein. J. Virol..

[90] Antonsson A., Johansson P.J.H. (2001). Binding of human and animal immunoglobulins to the IgG Fc receptor induced by human cytomegalovirus. J. Gen. Virol..

[91] Jenks J.A., Goodwin M.L., Permar S.R. (2019). The Roles of Host and Viral Antibody Fc Receptors in Herpes Simplex Virus (HSV) and Human Cytomegalovirus (HCMV) Infections and Immunity. Front. Immunol..

[92] Li F., Freed D.C., Tang A., Rustandi R.R., Troutman M.C., Espeseth A.S., Zhang N., An Z., McVoy M., Zhu H. (2017). Complement enhances in vitro neutralizing potency of antibodies to human cytomegalovirus glycoprotein B (gB) and immune sera induced by gB/MF59 vaccination. NPJ Vaccines.

[93] Britt W.J., Vugler L., Stephens E.B. (1988). Induction of complement-dependent and -independent neutralizing antibodies by recombinant-derived human cytomegalovirus gp55-116 (gB). J. Virol..

[94] Ohta A., Fujita A., Murayama T., Iba Y., Kurosawa Y., Yoshikawa T., Asano Y. (2009). Recombinant human monoclonal antibodies to human cytomegalovirus glycoprotein B neutralize virus in a complement-dependent manner. Microbes Infect..

[95] Da Costa X.J., Brockman M.A., Alicot E., Ma M., Fischer M.B., Zhou X., Knipe D.M., Carroll M.C. (1999). Humoral response to herpes simplex virus is complement-dependent. Proc. Natl. Acad. Sci. USA.

[96] Liszewski M.K., Kolev M., Le Friec G., Leung M., Bertram P.G., Fara A.F., Subias M., Pickering M.C., Drouet C., Meri S. (2013). Intracellular complement activation sustains T cell homeostasis and mediates effector differentiation. Immunity.

[97] Martin M., Leffler J., Smolag K.I., Mytych J., Bjork A., Chaves L.D., Alexander J.J., Quigg R.J., Blom A.M. (2016). Factor H uptake regulates intracellular C3 activation during apoptosis and decreases the inflammatory potential of nucleosomes. Cell Death Differ..

[98] Schaffler A., Buechler C. (2012). CTRP family: Linking immunity to metabolism. Trends Endocrinol. Metab..

[99] Tam J.C., Bidgood S.R., McEwan W.A., James L.C. (2014). Intracellular sensing of complement C3 activates cell autonomous immunity. Science.

